# Perspectives on Approaches in Psychiatry: A Resident Survey in Tunisia

**DOI:** 10.1192/j.eurpsy.2025.1938

**Published:** 2025-08-26

**Authors:** K. Njeh, A. Aissa

**Affiliations:** 1 Psychiatry A, Razi psychiatric hospital; 2 Hopital Razi, Manouba, Tunisia

## Abstract

**Introduction:**

The field of psychiatry encompasses a range of approaches that guide both clinical practice and training.

This study aims to explore residents’ views on these diverse approaches, identifying trends and preferences that could inform the development of psychiatric education and clinical practice in the region.

**Objectives:**

The objective of this study is to assess the perspectives of psychiatry and child psychiatry residents in Tunisia on different approaches to mental health care and to identify their preferences and attitudes towards evidence-based psychiatry.

**Methods:**

A cross-sectional survey was conducted among 31 psychiatry and child psychiatry residents in Tunisia through an online survey.

The survey utilized a combination of yes/no questions and gradual questions to gather data on residents’ perspectives regarding various psychiatric approaches.

Data collection was carried out throughout the month of july 2024.

**Results:**

This study included 31 participants with an average age of 28.1 years.

Less than half of the participants (45.2%) reported that they believe the brain is the sole source of mental disorders, with a notable proportion identifying as non-theist.

A majority of residents (80.5%) agreed that medicine should be grounded in the scientific method. However, only 14 participants agreed that “Evidence-based approaches should be the only approaches in psychiatry,” and 15 participants agreed that “The biological approach should be the primary focus in psychiatry.”

Most residents considered most approaches to be evidence-based. In contrast, the psychodynamic approach was less frequently viewed as evidence-based.

Nearly all residents (n=27) believed that an integrative approach is beneficial in psychiatric practice.

More than half of the participants (54.8%) rated their training in Evidence-Based Psychiatry as limited. Despite this, 24 participants reported that they often try to adhere to evidence-based guidelines when treating patients.

The main barriers to implementing Evidence-Based Psychiatry were identified as a lack of resources in psychiatric departments or hospitals (41.9%) and insufficient training (38.7%).

**Conclusions:**

The results indicate that while there is strong support for evidence-based medicine and the integration of multiple theoretical models, there are differing views on the predominance of specific approaches in clinical practice.

The majority of residents acknowledge the importance of grounding psychiatric practice in empirical evidence, yet there is less consensus on making evidence-based approaches the exclusive or primary focus.

Despite recognizing the benefits of integrating diverse approaches, many residents perceive limitations in their training and resources, which affect their ability to adhere fully to evidence-based practices.

The findings underscore the need for enhanced training in Evidence-Based Psychiatry and improved resources within psychiatric departments and hospitals.

**Disclosure of Interest:**

None Declared

